# Dynamic m^6^A mRNA Methylation Reveals the Role of METTL3/14-m^6^A-MNK2-ERK Signaling Axis in Skeletal Muscle Differentiation and Regeneration

**DOI:** 10.3389/fcell.2021.744171

**Published:** 2021-10-01

**Authors:** Shu-Juan Xie, Hang Lei, Bing Yang, Li-Ting Diao, Jian-You Liao, Jie-Hua He, Shuang Tao, Yan-Xia Hu, Ya-Rui Hou, Yu-Jia Sun, Yan-Wen Peng, Qi Zhang, Zhen-Dong Xiao

**Affiliations:** ^1^Vaccine Research Institute of Sun Yat-sen University, The Third Affiliated Hospital of Sun Yat-sen University, Guangzhou, China; ^2^Biotherapy Center, The Third Affiliated Hospital of Sun Yat-sen University, Guangzhou, China; ^3^Guangdong Provincial Key Laboratory of Malignant Tumor Epigenetics and Gene Regulation, Medical Research Center, Sun Yat-sen Memorial Hospital, Sun Yat-sen University, Guangzhou, China

**Keywords:** m^6^A, METTL3/14, MNK2, ERK signaling, skeletal muscle differentiation and regeneration

## Abstract

N^6^-methyladenosine (m^6^A) RNA methylation has emerged as an important factor in various biological processes by regulating gene expression. However, the dynamic profile, function and underlying molecular mechanism of m^6^A modification during skeletal myogenesis remain elusive. Here, we report that members of the m^6^A core methyltransferase complex, METTL3 and METTL14, are downregulated during skeletal muscle development. Overexpression of either METTL3 or METTL14 dramatically blocks myotubes formation. Correspondingly, knockdown of METTL3 or METTL14 accelerates the differentiation of skeletal muscle cells. Genome-wide transcriptome analysis suggests ERK/MAPK is the downstream signaling pathway that is regulated to the greatest extent by METTL3/METTL14. Indeed, METTL3/METTL14 expression facilitates ERK/MAPK signaling. Via MeRIP-seq, we found that MNK2, a critical regulator of ERK/MAPK signaling, is m^6^A modified and is a direct target of METTL3/METTL14. We further revealed that YTHDF1 is a potential reader of m^6^A on MNK2, regulating MNK2 protein levels without affecting mRNA levels. Furthermore, we discovered that METTL3/14-MNK2 axis was up-regulated notably after acute skeletal muscle injury. Collectively, our studies revealed that the m^6^A writers METTL3/METTL14 and the m^6^A reader YTHDF1 orchestrate MNK2 expression posttranscriptionally and thus control ERK signaling, which is required for the maintenance of muscle myogenesis and may contribute to regeneration.

## Introduction

Multiple layers of epigenetic regulation play essential roles in skeletal muscle development, and body movement in mammals requires a highly coordinated epigenetic regulatory network. Our previous studies clarified that DNA methylation and demethylation (Diao et al., 2021b), as well as noncoding RNAs, including miRNAs and lncRNAs (long noncoding RNAs), play pivotal roles in skeletal muscle differentiation (Huang et al., 2011; Xie et al., 2018; Liu et al., 2021; Tan et al., 2021). Additionally, RNA modifications have recently been recognized as novel epigenetic regulators. More than one hundred types of chemical modifications have been identified in cellular RNAs (Roundtree et al., 2017), among which N^6^-methyladenosine (m^6^A) is the one most extensively studied.

m^6^A RNA methylation is one of the most prevalent modifications of messenger RNAs (mRNAs) in cells, and its dysregulation has been linked to developmental dysplasia, cancer and other human diseases (Deng et al., 2018; Wang et al., 2018, 2019; Weng et al., 2018; Zhang M. et al., 2020). m^6^A is a reversible and dynamic RNA modification. High-throughput sequencing studies revealed that m^6^A modifications may affect thousands of genes in a given type of cell. m^6^A sites were found in the consensus RNA motif “RRACH” (R = A or G; H = A, U, or C), which is enriched near stop codons and in 3′ untranslated regions (UTRs) of mRNA (Meyer et al., 2012). The abundance and effects of m^6^A on RNA are determined by the dynamic interplay between methyltransferases (“writers,”) binding proteins (“readers”) and demethylases (“erasers”) (Yang et al., 2018; Shi et al., 2019). The m^6^A writer machinery is a methyltransferase complex including the m^6^A-METTL complex and the m^6^A-METTL-associated complex, which comprises methyltransferase-like 3 (METTL3)/methyltransferase-like 14 (METTL14) and Wilm’s tumor 1-associating protein (WTAP)/ZC3H13/VIRMA/HAKAI (Ping et al., 2014; Knuckles et al., 2018). METTL3 and METTL14 are the core m^6^A writers, and both contain methyltransferase domains and engage in cooperative functions, but only METTL3 is the catalytically active subunit; METTL14 plays a structural role critical for substrate recognition (Wang et al., 2016; Meyer and Jaffrey, 2017). m^6^A-mediated regulation is dependent on the recognition of m^6^A-binding proteins, which are represented by the YTH domain family (Meyer and Jaffrey, 2017) and IGF2BP proteins, which were identified after the YTH family (Huang et al., 2018). For instance, the cytoplasmic YTHDF proteins YTHDF1-3 were found to regulate the translation and decay of m^6^A-modified mRNA (Yang et al., 2015). The first study to explore the function of a YTH protein examined YTHDF2, which mediates the well-documented instability of m^6^A-containing mRNA and may interact only transiently with P-bodies to deliver mRNAs for decay. In contrast to YTHDF2, YTHDF1 was shown to promote translation without affecting mRNA stability (Meyer and Jaffrey, 2017). The m^6^A RNA modification can also be reversed by m^6^A demethylases, fat mass and obesity-associated protein (FTO) and a-ketoglutarate-dependent dioxygenase, alkB homolog 5 (ALKBH5) (Zhao et al., 2014; Yang et al., 2015). An interesting issue that requires further investigation is how these macromolecular complexes work in coordination and achieve precise control at multiple levels.

Our most recent study confirmed that the m^6^A key methyltransferase METTL3 is involved in the biogenesis of muscle-specific miRNAs (Diao et al., 2021a). This finding is consistent with a previous report indicating that METTL3 is sufficient to enhance miRNA maturation in a global and non-cell-type specific manner (Alarcón et al., 2015). Other studies have shown that m^6^A methylation is involved in muscle formation and maintaining muscle homeostasis, as well as in multifaceted functions in musculoskeletal disorders (Zhang W. et al., 2020). For example, Kudou et al. (2017) reported that the myogenic potential is maintained partly by the Mettl3-mediated stabilization of processed MyoD mRNA through m^6^A modification of the 5′ UTR during proliferative phases. Gheller et al. (2020) showed that global m^6^A levels were increased after skeletal muscle injury at a time point corresponding to rapid muscle stem cell (MuSC) proliferation, and global m^6^A levels in primary mouse myoblasts or C2C12 myoblasts were shown to increase during proliferation and to decline during differentiation *in vitro*. METTL3 knockdown affected MuSC engraftment after primary transplantation (Gheller et al., 2020). However, the key downstream targets and the underlying mechanism still need to be fully elucidated. Recently, Zhang X. et al. (2020) demonstrated that knockdown of METTL14, another m^6^A methyltransferase, inhibited the differentiation and promoted the proliferation of C2C12 myoblasts. Lin et al. (2020) found that METTL3 was upregulated in adipose-derived stem cells undergoing vascular smooth muscle differentiation induction. Overall, these studies suggest that the exact functions of METTL3 and METTL14 in skeletal muscle development are still unclear, especially the underlying molecular mechanism, which needs to be elucidated.

Here, we report a significant decrease in the m^6^A methylation level during myogenesis, which was accompanied by a decline in the expression of the key methyltransferases METTL3 and METTL14. Gain-of-function and loss-of-function studies revealed that METTL3/14 significantly inhibited the differentiation of skeletal muscle cells *in vitro*. Coupling RNA-seq and MeRIP-seq, we found that METTL3/14 could activate ERK/MAPK signaling and thus impair skeletal muscle differentiation by directly targeting MAP kinase-interacting kinase 2 (MKNK2, MNK2). Further results suggested that YTHDF1 is a critical reader for MNK2 because it regulated its protein levels without affecting mRNA levels. Furthermore, we found that METTL3/14-MNK2 axis favors the early stage muscle regeneration after acute injury. Our study revealed that a decrease in m^6^A methylation was required for skeletal muscle differentiation, but m^6^A was a positive regulator for the early stage of muscle regeneration after injury, suggesting that METTL3/14 regulates skeletal muscle differentiation and regeneration by modifying MNK2 via m^6^A.

## Materials and Methods

### Animal Studies

Male C57BL/6J mice were purchased from GemPharmatech Co., Ltd. (Nanjing, China). Mice were housed in the animal facility and had free access to water and standard rodent chow. All animals care and surgical procedures were approved by the Animal Ethics Committee at The Third Affiliated Hospital of Sun Yat-sen University.

Fetal (E12.5, E15.5, and E18.5) and postnatal (1, 2, and 8 weeks) hind limb muscles were isolated for RNA and protein extraction.

For mouse muscle injury and regeneration experiment, tibialis anterior (TA) muscles of 6-week-old male mice were injected with 25 μl of 10 μM cardiotoxin (CTX, Merck Millipore, 217503), 0.9% normal saline (Saline) were used as control. The regenerated muscles were collected at day 1, 3, 5, and 10 post-injection. TA muscles were isolated for Hematoxylin and eosin staining or frozen in liquid nitrogen for RNA and protein extraction.

### Isolation of Muscle Stem Cells

MuSCs were FACS-sorted from hind limb muscles of postnatal 1-week mice as previously described (Xie et al., 2018). Briefly, mechanical and enzymatic dissociation of cells, released resident mononucleated cells, then followed by antibody CD45 (PE/Cy7-CD45, eBioscience, 25-0451-82), CD31 (FITC-CD31, BioLegend, 102506), Sca1 (V450-Sca1, BD, 560653) and VCAM1 (efluor660-VCAM1, eBioscience, 50-1061-80) staining and FACS isolation. Collection of the cells those were positive for VCAM1 expression and negative for CD31, CD45, and Sca1.

### Cell Culture

HEK-293T and mouse skeletal muscle C2C12 cells were purchased from the Cellular Library of the National Collection of Authenticated Cell Cultures (Shanghai, China) and cultured in growth medium (GM), which consisted of Dulbecco’s Modified Eagle’s Medium (DMEM, Gibco) supplemented with 10% fetal bovine serum (FBS, Life Technology), 100 U/ml penicillin, and 100 μg/ml streptomycin (1 × penicillin–streptomycin). The sorted MuSCs were cultured in GM supplemented with 2.5 ng/mL bFGF (Life Technology). When MuSCs or C2C12 cells grow up to about 90% confluency, the GM was subsequently replaced with differentiation medium (DM), which was DMEM containing 2% horse serum (HyClone).

To inhibit the activity of the ERK signaling pathway, C2C12 cells were treated with 10 or 20 μM PD0325901 (PD, Sigma-Aldrich), respectively, and DMSO (Sigma-Aldrich) was used as the negative control.

### Western Blot and Antibodies

Cells or homogenized tissue were lysed in ice-cold RIPA buffer containing 1 × protease inhibitor cocktail and phosphatase inhibitor (Roche). Equivalent total protein extracts were separated by SDS-PAGE and transferred to nitrocellulose membrane (GE Healthcare Life science, Germany). After that, membranes were blocked with 5% bovine serum albumin (BSA) for 1 h at room temperature. Primary antibodies were incubated and a horseradish peroxidase-conjugated secondary antibody was followed. The following antibodies were used in this study: antibodies for MEF2C (#5030), p-ERK (#9101S), ERK (#9102S), p-AKT (Thr308, #13038), p-AKT (Ser473, #4060) and GAPDH (#2118) were obtained from Cell Signaling Technology. Antibodies for MyoD (sc-760), WTAP (sc-374280) and MNK2 (sc-271559) were obtained from SantaCruz. Antibodies for METTL3 (#15073-1-AP) and Vinculin (#26520-1-AP) were obtained from Proteintech. Antibodies for ALKBH5 (ab174124) and Ki67 (ab15580) was obtained from Abcam. Antibodies for MHC (MAB4470), METTL14 (HPA038002), FTO (#597-FTO) and GFP (AE012) were obtained from R&D, Sigma, PhosphoSoklutions and ABclonal, respectively. Horseradish peroxidase-conjugated secondary antibodies were used to detect the primary antibodies. Immunoreactivities were determined using the ECL method (Advansta). The gray value of each protein band was quantified using ImageJ software.

### Plasmids Construction and Stable Cell Generation

cDNAs of mouse METTL3 and METTL14 were subcloned to pKD-CMV-MCS-EF1-PURO (pKD) vector by Gibson Assembly, pKD-GFP were subcloned as negative control. The gRNAs downstream transcription start sites used to guide the fusion of inactive Cas9 (dCas9) to the Krüppel-associated box (KRAB) repressor. The shRNA oligonucleotides were annealed and cloned into pLKO.1-TRC plasmid. Cignal 45-pathway reporter arrays, the ERK pathway reporter which measure the activity of the EKR pathways were purchased from Qiagen (Qiagen, Hilden, Germany). To generate stable cells, lentivirus were produced in 293T cells by transfecting these lentiviral vetors respectively with psPAX.2 and pMD2.G. Forty eight hours later, lentiviruses were collected, filtered, and infected target cell lines. Stable cells were selected by puromycin antibiotic.

All primers for cloning were listed in Supplementary Table 1.

### RNA Oligonucleotides and Transient Transfection

Small interfering RNA (siRNA) targeting YTH family (si-YTHDF1, si-YTHDF2, si-YTHDF3, and si-YTHDC1), and corresponding negative control siRNA (si-NC) were obtained from GenePharma Co., Ltd. (Suzhou, China). The cells were reverse transfected using Lipofectamine 2000 according to the manufacturer’s protocol. A final concentration of 100 nM siRNAs was used for each transfection.

The sequences of the siRNAs were listed in Supplementary Table 2.

### RNA Extraction and Quantitative RT-PCR Analysis

Total RNA was extracted from cells or tissues with TRIzol reagent (Invitrogen). First-strand cDNA for PCR analyses was synthesized with a PrimeScript RT reagent kit (Takara), quantitative real-time PCR was performed using 2 × ChamQ Universal SYBR qPCR Master Mix (Vazyme). GAPDH gene was used as endogenous control. Expression values of RNA were calculated using the comparative Ct method. Results were presented as Mean ± SD.

All primers for qPCR were listed in Supplementary Table 1.

### m^6^A-RIP qPCR

m^6^A methylated RNA immunoprecipitation was performed as we previously described (Diao et al., 2021a). Briefly, 200 μg total RNA were incubated with 8 μg m^6^A antibody (#202003, Synaptic Systems, Germany) for 2 h on a rotating wheel at 4°C. Then, 50 μL protein A/G magnetic beads were added to reaction mixtures for 2 h on a rotating wheel at 4°C. After washed three times, beads were incubated with DNase I at 37°C for 30 min and were then digested by Proteinase K for about 2 h at 37°C with rotation. MicroElute RNA CleanUp Kit (Omega) was used for RNA purification. Purified RNA was reverse transcribed and was quantified by real-time RT-PCR.

### m^6^A Dot Blot Assay

Dot blot was performed as previously described with minor modification (Chen et al., 2015). Briefly, appropriate number of cells was and harvested avoiding RNA degradation, then purified by Dynabeads mRNA purification kit (Invitrogen, #61012). RNA samples were quantified and denatured at 95°C for 3 min, then cooled down on ice for 2 min. Samples were spotted on the membrane (Amersham Hybond-N^+^, GE) and air dry for 5 min, followed by UV-crosslink (2 auto-crosslink, 150 mJ/cm^2^ UV Stratalinker, STRATAGENE). Membranes were washed in TBST and dyed in Methylene-blue (Sigma-Aldrich) as quantitative control. Then blocked in 5% non-fat dry milk in TBST for 2 h at room temperature, incubated with m^6^A antibodies (1:1,000, Synaptic Systems, 202-003) for overnight at 4°C. After 3 time washes, membranes were incubated with HRP linked secondary anti-rabbit IgG antibody (1:5,000, CST, #7074) for 1 h at room temperature. Signals were detected with ECL Plus Western Blotting Reagent Pack (GE Healthcare). The dots were quantified by Image J.

### Immunofluorescence

Cells were fixed in 4% formaldehyde for 30 min at room temperature prior to cell permeabilization with 0.1% Triton X-100 at 4°C for 10 min. Then, cells were blocked with 2.5% BSA in PBS (Beyotime) for 2.5 h. After blocking, cells were incubated with an anti-MHC antibody overnight at 4°C. The cells were washed 3 times with PBS for 10 min and then incubated with an Alexa Fluor 594-conjugated (Invitrogen) secondary antibody for 1 h. Nuclei were labeled with Hoechst (Invitrogen) for 10 min at a concentration of 0.5 μg/mL, and fluorescence signals were acquired and analyzed by laser scanning confocal microscopy (Zeiss).

### Dual-Luciferase Reporter Assays

For analysis of the Cignal finder pathway reporter arrays, transient transfections were performed in C2C12 cells stably overexpressed GFP or METTL3/14 as previously reported (Diao et al., 2021b). 50 ng of Cignal pathway reporter and standard negative control were transfected using lipofectamine 2000 reagent (Invitrogen) according to the manufacturer’s protocol. Firefly and Renilla luciferase activities were consecutively measured at 48 h after transfection using the Dual-Luciferase Reporter Assay kit (Promega).

### MeRIP Sequencing

Total RNA was isolated and purified using TRIzol reagent (Invitrogen) following the manufacturer’s procedure. The RNA amount and purity of each sample was quantified using NanoDrop ND-1000 (NanoDrop). The RNA integrity was assessed by Bioanalyzer 2100 (Agilent) with RIN number > 7.0, and confirmed by electrophoresis with denaturing agarose gel. Poly (A) RNA is purified from 50 μg total RNA using Dynabeads Oligo (dT) 25-61005 (Thermo Fisher Scientific). Then the poly (A) RNA was fragmented into small pieces using Magnesium RNA Fragmentation Module (NEB). Then the cleaved RNA fragments were incubated with m^6^A-specific antibody (#202003, Synaptic Systems, Germany). Then the IP RNA was reverse-transcribed to create the cDNA by SuperScript^TM^ II Reverse Transcriptase (Invitrogen, cat. 1896649), and fowling to synthesize U-labeled second-stranded DNAs. Then ligation with the adapter to the A-tailed fragmented DNA, the ligated products are amplified with PCR and performed the 2 × 150 bp paired-end sequencing (PE150) on an Illumina NovaSeq^TM^ 6000 (LC-Bio Technology Co., Ltd., Hangzhou, China) following the vendor’s recommended protocol.

### Statistical Analysis and Data Mining

For meRIP-seq, Fastp software^1^ was used to remove the reads that contained adaptor contamination, low quality bases and undetermined bases with default parameter. Then sequence quality of IP and Input samples were also verified using fastp. We used HISAT2^2^ to map reads to the reference genome (GRCm38, Ensembl) and kept unique mapped reads via samtools based on mapping quality larger than 30. Mapped reads of IP and input libraries were provided for R package exomePeak,^3^ which identifies significant m^6^A peaks and differential peaks, FDR ≤ 0.05 was considered significant. Bigwig format was produced via deeptools, as to be used for visualization on the IGV software.^4^ HOMER^5^ was used for *de novo* motif discovery based on top 1k most-enrichment peaks. Called peaks were annotated by intersecting with gene architecture using bedtools and custom python script. m^6^A metagene plot was plotted using Guitar package.

For mRNA-seq, clean fastq reads were aligned to mouse genome (Ensembl GRCm38) using hisat2 software. Raw gene read counts were calculated using featureCounts software based on Ensembl gene annotation (version 102), then values were normalized as RPKM (Reads per kilobase of exon per million reads mapped) using fpkm function in DESeq2 package. The differentially expressed genes were identified using DESeq2 package, genes with FDR (false discovery rate) ≤ 0.05 were considered statistically significant. GO and KEGG analyses were analyzed using clusterProfiler, *p* ≤ 0.05 were considered statistically significant. Heatmap was plotted using pheatmap package. Circos plot was plotted using Circos software.

Data are presented as the mean ± SD for three independent experiments unless otherwise noted in the figure legends. The statistical significance of difference between two means was calculated with the Student’s *t*-test (^∗^*p* < 0.05, ^∗∗^*p* < 0.01, ^****^p < 0.001, ^****^p < 0.0001), using Prism 9.0 (GraphPad Software).

## Results

### Down-Expression of Mettl3 and Mettl14 Are Required for Skeletal Muscle Development

**FIGURE 1:**
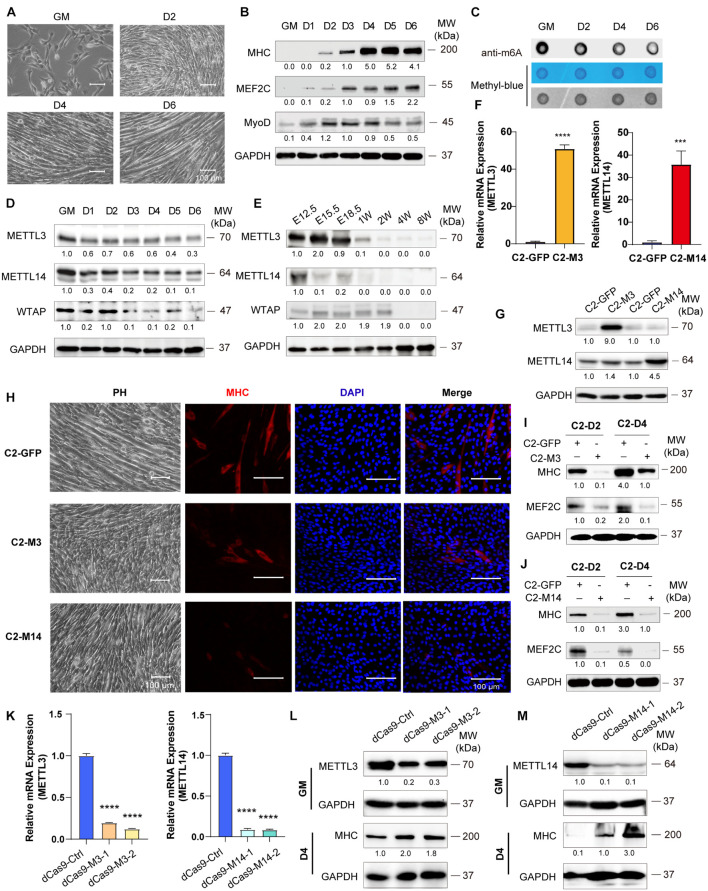
Down-expression of Mettl3 and Mettl14 are required for skeletal muscle development. **(A)** Phenotype of differentiation C2C12 myoblasts in growth medium (GM) or day2 (D2), day4 (D4) or day6 (D6) in differentiation medium. **(B)** Western blot analysis of skeletal muscle differentiation markers MHC, MEF2C and MyoD of differentiation C2C12 myoblasts. **(C)** RNA dot blot assay of m^6^A methylation level of differentiation C2C12 myoblasts. **(D)** Western blot analysis of core components of m^6^A methyltransferases METTL3, METTL14 and WTAP in differentiating C2C12 myoblasts. **(E)** Western blot analysis of core components of m^6^A methyltransferases METTL3, METTL14 and WTAP during developing mouse embryos muscle differentiation. Mouse hind limb muscles were isolated from seven time points: E12.5, E15.5, E18.5 (embryos), postnatal 1 week, postnatal 2 weeks, postnatal 4 weeks, and postnatal 8 weeks (adult). The expression levels of METTL3 and METTL14 in the indicated METTL3- or METTL14- overexpressing C2C12 cells were determined by **(F)** Real-time PCR and **(G)** western blot. **(H)** Cell phenotype of the indicated C2C12 stable cells for differentiation 3 days. And immunofluorescent microscopy analysis of the expression of myogenic marker MHC in the indicated C2C12 stable cells for differentiation 3 days. The Scale bar indicated as 100 μm. The expression of myogenic marker MHC and MEF2C in the indicated METTL3- **(I)** or METTL14- **(J)** overexpressing C2C12 stable cells upon differentiation for 2 or 4 days were detected by western blot. **(K)** Real-time PCR analysis of the RNA expression levels of METTL3 or METTL14 in the indicated METTL3/14 knockdown C2C12 stable cell lines. METTL3 or METTL14 were determined by western blot in indicated METTL3 **(L)** or METTL14 **(M)** knockdown C2C12 stable cell lines in GM, and myogenic marker MHC was determined in D4. Quantitative data was represented as Mean ± SD. ***p < 0.001, ****p < 0.0001.

To examine skeletal muscle differentiation, mouse C2C12 myoblasts were used to study myogenesis *in vitro*. We expanded C2C12 myoblasts in the presence of serum and initiated differentiation by serum withdrawal. The cell phenotype changed greatly, as described in our previous reports (Xie et al., 2018; Tan et al., 2021; Figure 1A). The differentiation of these cells was confirmed by the myogenic markers MHC, MEF2C and MyoD (Figure 1B). RNA dot blot assay was performed to investigate the dynamics of m^6^A RNA modification during myogenesis. We found that the levels of m^6^A marks were decreased during C2C12 cell differentiation. A significant downregulation of m^6^A levels was observed after serum withdrawal (Figure 1C and Supplementary Figure 1A). These results indicated that m^6^A methylation was reduced during skeletal muscle differentiation. Next, we wondered whether the decrease in m^6^A RNA modification was due to the altered expression of writers or erasers. We profiled the core components of m^6^A methyltransferases and demethylases. Interestingly, RNA and protein expression of METTL3/14 and WTAP were all significantly downregulated (Figure 1D and Supplementary Figure 1B) and negatively correlated with MHC and MEF2C expression (Figure 1B). And similar protein expression of these genes were shown in primary mouse skeletal muscle cells (Supplementary Figure 1C). While, the demethylases FTO and ALKBH5 had opposite changes during C2C12 differentiation (Supplementary Figure 1D), and the upregulation of FTO is consistent with previous report (Wang et al., 2017). To confirm the changes in the core m^6^A components during skeletal muscle development *in vivo*, we further profiled the expression of these genes in the hind limb muscle of developing mouse embryos. Consistent with the C2C12 cell mimic skeletal muscle differentiation results, METTL3/14 and WTAP protein levels were drastically downregulated (Figure 1E). The real-time PCR results also showed that all m^6^A modification methyltransferase genes were downregulated during mouse skeletal muscle development (Supplementary Figure 1E). These results implied that the downregulation of m^6^A was mainly due to the downregulation of its core methyltransferases, METTL3/14.

Given the robust functional roles of METTL3 and METTL14 methyltransferases in m^6^A RNA modification, as well as the equivocal METTL3 and METTL14 function in skeletal muscle homeostasis (Gheller et al., 2020; Zhang X. et al., 2020), we overexpressed METTL3 or METTL14 in C2C12 cells, and GFP-overexpressing cells were used as negative controls. The real-time PCR and western blot results validated the overexpression of METTL3/14 (Figures 1F,G). When METTL3 or METTL14 was overexpressed, the formation of myotubes was suppressed, which was confirmed by myosin immunofluorescence staining (Figure 1H). The western blot assays clearly showed that the expression of the differentiation marker MHC and that of the muscle-specific transcription factor MEF2C were decreased upon overexpression of METTL3/14 (Figures 1I,J and Supplementary Figures 2A,B). The suppression of MHC and MEF2C by overexpressed METTL3/14 was further confirmed by real-time PCR assays (Supplementary Figures 2C,D).

To complement loss-of-function experiments, we designed two gRNAs downstream of transcription start sites and used them to guide the fusion of inactive Cas9 (dCas9) to the Krüppel-associated box (KRAB) repressor to inhibit the transcription of METTL3 or METTL14 in C2C12 cells. As shown in Figure 1K, the mRNA levels of Mettl3 or Mettl14 were greatly reduced compared to those of the control gRNA. Correspondingly, the protein levels of METTL3 and METTL14 were decreased (Figures 1L,M). Four days after the cell growth medium (GM) was switched to differentiation medium (DM), the protein levels of the differentiation marker MHC were clearly upregulated when METTL3 or METTL14 was knocked down (Figures 1L,M), validating the finding showing that METTL3 or METTL14 could inhibit the differentiation of C2C12 cells. Taken together, all these results suggested that down-expression of Mettl3 and Mettl14 are required for skeletal muscle development.

### METTL3/14 Blocks the Differentiation of Primary Mouse Skeletal Muscle Cells

**FIGURE 2:**
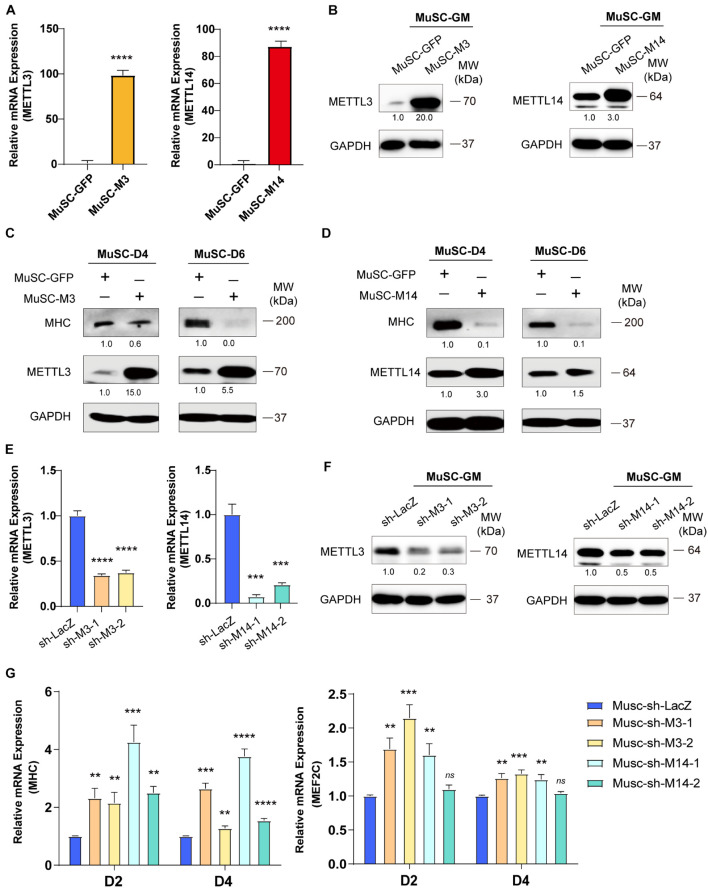
METTL3/14 blocks the differentiation of primary mouse skeletal muscle cells. The expression of METTL3 and METTL14 in the indicated METTL3- or METTL14- overexpressing MuSCs were determined by **(A)** Real-time PCR and **(B)** western blot. The expression of myogenic marker MHC in METTL3- **(C)** or METTL14- **(D)** overexpressing MuSCs upon differentiation for 4 or 6 days were detected by western blot. **(E)** Real-time PCR analysis of the RNA expression levels of METTL3 or METTL14 in the indicated METTL3/14 knockdown MuSC stable cells. **(F)** METTL3 or METTL14 were determined by western blot in indicated METTL3/14 knockdown MuSC stable cells. **(G)** Real-time PCR analysis of the RNA expression levels of myogenic marker MHC and MEF2C were determined in D2 and D4. Quantitative data was represented as Mean ± SD. **p < 0.01, ***p < 0.001, ****p < 0.0001, *ns* stands for not statistically significant.

To confirm the anti-differentiation functions METTL3 and METTL14, we isolated MuSC primary mouse skeletal muscle cells and infected them with lentiviruses encoding METTL3 or METTL14. Real-time PCR and western blot assays validated the success of METTL3/14 overexpression (Figures 2A,B). As determined by western blot assay, the MHC protein levels were clearly decreased when METTL3 or METTL14 was overexpressed (Figures 2C,D). The decrease in the differentiation markers MHC and MEF2C was also confirmed by real-time PCR assays (Supplementary Figure 3). These results suggested that METTL3/14 could repress the differentiation of primary mouse skeletal muscle cells.

To confirm our loss-of-function study, we knocked down METTL3 or METTL14 in MuSC primary mouse skeletal muscle cells. METTL3 and METTL14 were successfully knocked down, as shown by the significant decreases in mRNA and protein levels (Figures 2E,F). Via real-time PCR, we found that the mRNA levels of MHC and MEF2C were significantly upregulated (Figure 2G), validating the finding showing that METTL3/14 could repress the differentiation of mouse primary skeletal muscle cells. Thus, knocking down METTL3/14 promoted the differentiation of C2C12 and primary mouse skeletal muscle cells.

Taken together, our findings revealed that METTL3/14 inhibited muscle differentiation. Considering our results with those of previous studies (Gheller et al., 2020), we clarified that the m^6^A methyltransferases METTL3 and METTL14 have a common myogenetic suppressor function during skeletal muscle differentiation.

### METTL3/14 Activates ERK Signaling in C2C12 Cells

To delineate the molecular implications of m^6^A methylation during skeletal muscle differentiation, we profiled genome-wide gene expression using RNA-seq when METTL3 or METTL14 was overexpressed in C2C12 cells (Supplementary Table 3).

**FIGURE 3:**
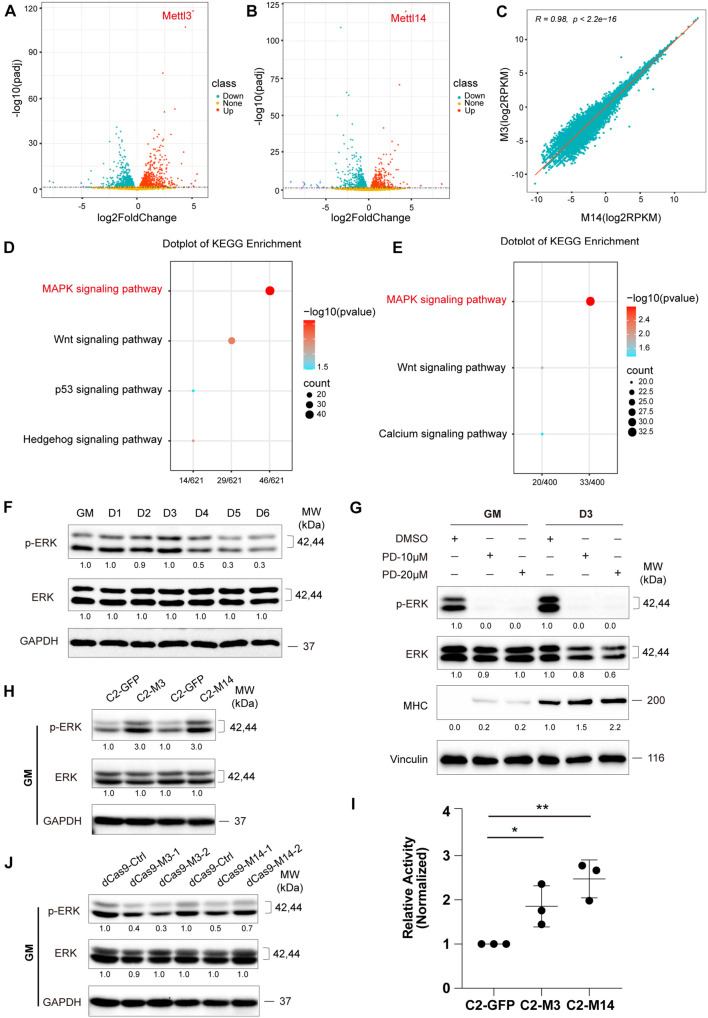
METTL3/14 activates ERK signaling in C2C12 cells. Volcano plot of differentially expressed genes in METTL3- **(A)** or METTL14- **(B)** overexpressing C2C12 cells in GM condition. **(C)** Correlation analysis of RNA profiles of METTL3- and METTL14- overexpressing C2C12 cells in GM condition. KEGG enrichment analysis of METTL3- **(D)** or METTL14- **(E)** overexpressing C2C12 cells. **(F)** Western blot analysis of ERK and its phosphorylated forms p-ERK of differentiation C2C12 myoblasts. **(G)** Western blot analysis of ERK, p-ERK and myogenic marker MHC in C2C12 cells that were treated with different dose of PD0325901 (PD) or DMSO for 48 h in growth medium (GM) or for 48 h in growth medium then shifted to differentiation medium for 3 days (D3). DMSO as negative control. **(H)** Western blot analysis of ERK and p-ERK in METTL3- or METTL14- knockdown C2C12 cells in GM condition. **(I)** The pathway assay revealed METTL3/14 affected the activity of ERK signaling. This examination was carried out in GM condition and the results were normalized to GFP overexpressing cells. Quantitative data was represented as Mean ± SD. The statistical significance of difference between two means was calculated with the *t*-test, *p < 0.05, **p < 0.01. **(J)** Western blot analysis of ERK and p-ERK in METTL3 or METTL14 knockdown C2C12 stable cell lines.

First, we analyzed the RNA-seq data of METTL3- or METTL14-overexpressing cells compared with those of the GFP negative control group, and the results are shown in Figures 3A,B. A panel of genes was significantly differential expressed when METTL3 or METTL14 was overexpressed (Supplementary Tables 4, 5). Furthermore, there was a perfectly positive correlation between the overall expression profile of these two groups (*R* = 0.98, *p* < 2.2e-16). These data verified the consistency of the METTL3 and METTL14 functions (Figure 3C). Next, we performed gene ontology analysis of the differentially expressed genes, and a KEGG pathway analysis revealed that the MAPK signaling pathway was the main significant signaling pathway, as indicated in both the METTL3 and METTL14 overexpression data (Figures 3D,E and Supplementary Tables 6, 7).

Many genes participate in the signal transduction of the MAPK cascade, and the ERK/MAPK signaling pathway has been reported to be involved in promoting the proliferation and inhibited differentiation of skeletal muscle (Michailovici et al., 2014; Yohe et al., 2018). We profiled ERK/MAPK signaling activity during the differentiation of C2C12 cells by detecting the phosphorylation of ERK via western blotting. As shown in Figure 3F, the ERK phosphorylation levels were downregulated when the growth medium of the C2C12 cells was switched to differentiation medium, suggesting that the activity of ERK signaling decreased during the differentiation of C2C12 cells. We chose AKT signaling as a negative control pathway and then tested the two phosphorylated forms of AKT, p-ATK at Thr308 and p-ATK at Ser473. As expected, the expression of these two phosphorylated forms of AKT did not change during muscle differentiation (Supplementary Figure 4A). To assess the role of ERK signaling in C2C12 cell differentiation, we treated C2Cl2 cells with the ERK-specific inhibitor PD0325901. ERK signaling activity was clearly inhibited, as indicated by ERK phosphorylation levels, while C2C12 cell differentiation was induced, elevating the expression of the myogenic markers MHC and MEF2C (Figure 3G and Supplementary Figure 5), indicating that ERK signaling could repress the differentiation of C2C12 cells. Interestingly, overexpression of METTL3 or METTL14 increased ERK phosphorylation levels and activated ERK signaling (Figures 3H,I), while knockdown of METTL3 or METTL14 showed the opposite effects (Figure 3J), suggesting that METTL3 or METTL14 may repress the differentiation of C2C12 cells by promoting ERK signaling activity. In the negative control pathway, the phosphorylated AKT proteins did not change when METTL3 or METTL14 expression was altered (Supplementary Figures 4B,C).

### MeRIP Sequencing Reveals MNK2 as an m^6^A-Enriched Transcript

**FIGURE 4:**
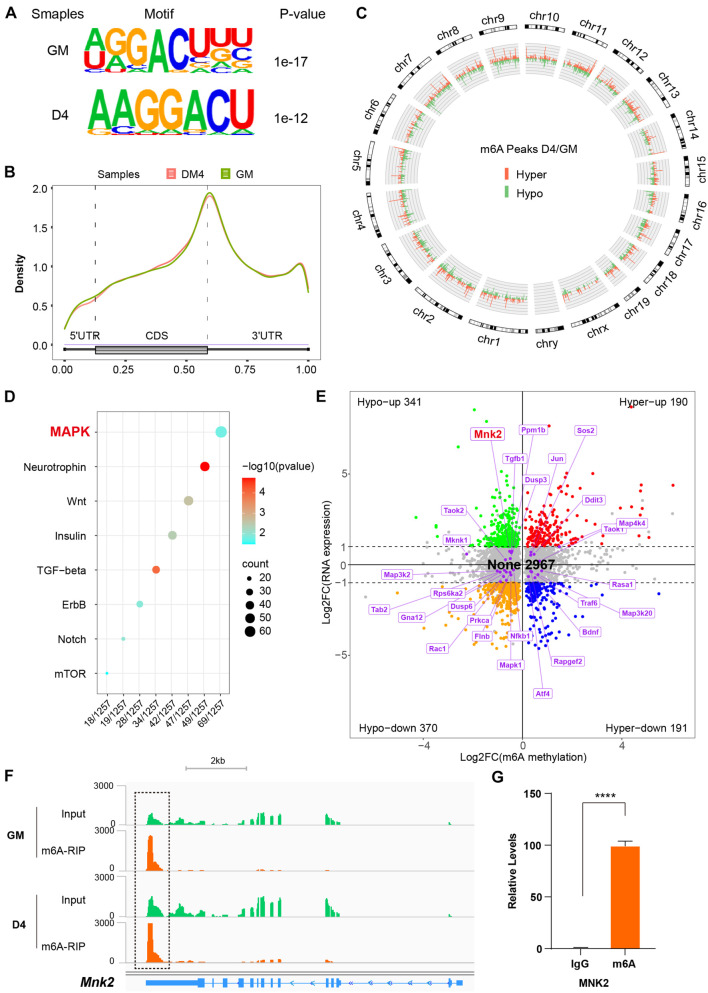
MeRIP sequencing reveals MNK2 as an m^6^A-enriched transcript. **(A)** Top consensus motif identified with m^6^A peaks before (GM) and after differentiation (D4). **(B)** Metagene profiles of enrichment of all m^6^A peaks across mRNA transcriptome. **(C)** Circos analysis of the distribution of the significantly changed m^6^A peaks across the whole mouse genome. The statistical significance was FDR ≤ 0.05 (light green indicates downregulated peaks, and light orange indicates upregulated peaks). **(D)** KEGG enrichment analysis of signaling pathway for differentially m^6^A genes during myogenesis. **(E)** Distribution of genes with a significant change in both the m^6^A methylation and RNA expression levels before (GM) and after differentiation (D4), different colors were used to identified of representative genes, the none changed curve of RNA expression were defined as below twofold. **(F)** Integrative Genomics Viewer (IGV) plots showing methylation levels of Mnk2 gene upon differentiation (light green indicates input data, yellow orange indicates RIP data). **(G)** Real-time PCR detection of Mnk2 expression in immunoprecipitated RNAs. IgG Immunoprecipitation was used as negative control. Quantitative data was represented as Mean ± SD. ****p < 0.0001.

Given the recent observations that METTL3/14 regulates target function via m^6^A RNA modification, we performed MeRIP-seq to decode the underlying m^6^A-RNA mechanisms in C2C12 cells. Two representative stages were selected: GM, in which myoblasts were cultured in growth medium corresponding to an undifferentiated state, and D4, in which cells were first grown to about 90% confluency and then replaced differentiation medium for 4 days, at which time the cells differentiated into obvious myotubes. We obtained over 20 thousand unique peaks from each MeRIP-seq sequencing library (FDR ≤ 0.05, Supplementary Tables 8–10). The *de novo* motif predicted by HOMER showed that the m^6^A sites of all the samples were highly concordant within the typical m^6^A motif “RRACH” (Figure 4A). The stability of m^6^A-tagged transcripts showed non-significant difference between the GM and D4 groups (Supplementary Figure 6A). Consistent with previous studies (Meyer et al., 2012), m^6^A modifications were not randomly distributed in mRNA sequences but were mainly enriched in the 3′UTR (Figure 4B and Supplementary Figures 6B,C). Furthermore, we analyzed the m^6^A peaks in the whole transcriptome, and the results showed 1848 unique peaks with increased m^6^A modification and 3501 unique peaks with reduced m^6^A modification (Figure 4C and Supplementary Table 11). This result indicated lower methylation in DM4 peaks. These data were consistent with the dot blot results of C2C12 mimic differentiation.

To determine which m^6^A gene cascades are involved in the skeletal muscle differentiation process, we analyzed the differentially expressed m^6^A peaks. The KEGG analysis showed that the MAPK signaling pathway was the major signaling pathway. As shown in Figure 4D, 69 of 1257 genes were enriched in the MAPK pathway (Supplementary Table 12).

To investigate the effects of altered m^6^A modification on RNA expression, we analyzed the RNA-seq data together with the MeRIP-seq data. Among 4059 significant changed m^6^A-hyper and m^6^A-hypo genes, 531 genes showed increased RNA levels (Hypo-up plus Hyper-up), and 561 genes showed reduced RNA levels (Hypo-down plus Hyper-down). Interestingly, 2967 genes showed negligible changes in RNA levels (None) (Figure 4E). These results indicated that the more than half (2,967 out of 4,059) genes contain m^6^A marks were not affect their RNA expression significantly in our study model. Thus, we checked the 69 significantly m^6^A-modified MAPK factors in the RNA-seq data, and 45 out of 69 m^6^A-modified MAPK factors were found to be significantly expression in the D4 stage vs. the GM stage RNA-seq data, and to our surprise, 27 genes showed no change in corresponding RNA expression, as shown in Figure 4E. One of the highly m^6^A-enriched transcripts, MNK2, was of particular interest, as it has been reported to play an essential role in MAPK signaling and is a well-known regulator of ERK signaling (Waskiewicz et al., 1997; Ueda et al., 2010). The findings prompted us to analyze the m^6^A target MNK2 in the MAPK subgroup. Our MeRIP-seq data revealed one clear m^6^A peak around the 3′UTR of *MNK2* mRNA (Figure 4F). To confirm that MNK2 was enriched in m^6^A marks, we used an antibody against m^6^A and performed RNA immunoprecipitation followed by real-time PCR. As shown in Figure 4G, compared to that in the IgG control, MNK2 RNA was significantly enriched in the m^6^A group, indicating that the MNK2 transcript was m^6^A enriched. Taken together, our data showed that a large number of RNAs which modified by m^6^A with slightly change in their RNA expression levels, and within that MNK2 is an m^6^A-enriched transcript involved in skeletal muscle differentiation.

### MNK2 Is a YTHDF1-Dependent Downstream Target of METTL3/14

**FIGURE 5:**
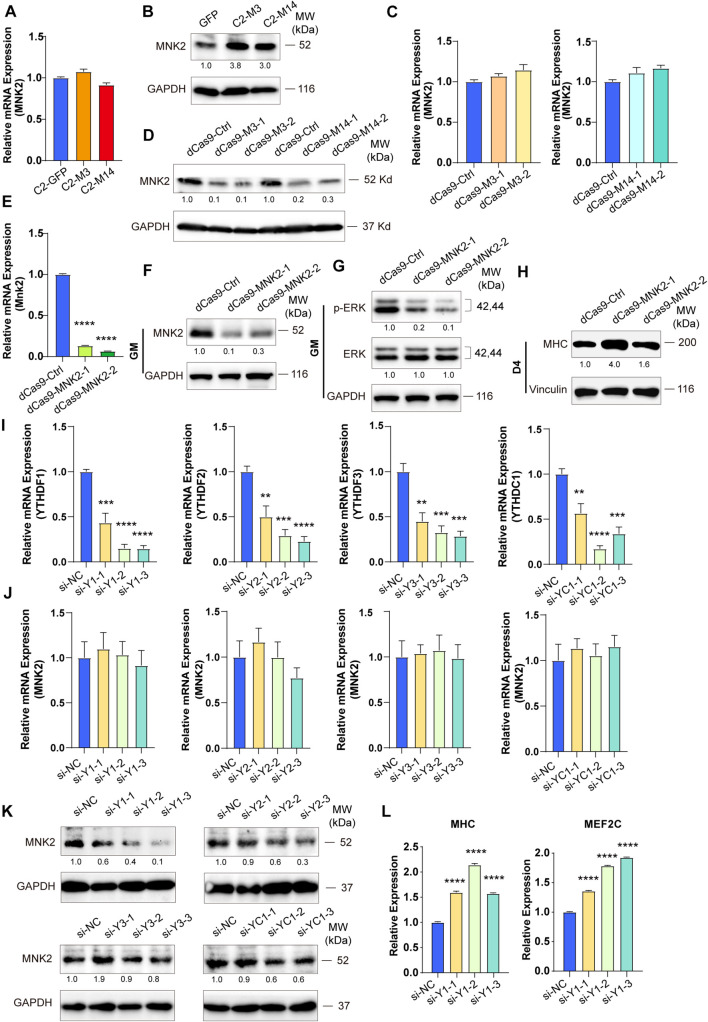
MNK2 is a downstream target of METTL3/14 via YTHDF1-dependent manner. METTL3/14 doesn’t affect the RNA expression level of MNK2. Real-time PCR analysis of the expression of MNK2 in the indicated METTL3- or METTL14- overexpressing **(A)** or knockdown **(C)** C2C12 cells. METTL3/14 promotes the protein expression of MNK2. MNK2 was determined by western blot in indicated METTL3- or METTL14- overexpressing **(B)** or knockdown **(D)** C2C12 cells. **(E)** Real-time PCR analysis of the expression of MNK2 in the MNK2 knockdown C2C12 stable cell lines. **(F)** MNK2 was determined by western blot in the MNK2 knockdown C2C12 stable cell lines in GM condition. **(G)** MNK2 affects protein expression of ERK signaling. This examination were carried out in GM condition and Vinculin as internal control. **(H)** MNK2 accelerates the protein expression of myogenic marker MHC. This examination were carried out in D4 condition and Vinculin as internal control. **(I)** Real-time PCR assays of each reader in the indicated YTH siRNA or siNC treatment. **(J)** si-YTH doesn’t affect the RNA expression level of MNK2. **(K)** The effects of si-YTH on the protein expression level of MNK2. **(L)** si-YTHDF1 accelerates RNA expression of MHC and MEF2C. Quantitative data was represented as Mean ± SD. **p < 0.01, ***p < 0.001, ****p < 0.0001.

To test whether MNK2 is a functional target of METTL3 and METTL14, we detected the expression of MNK2 when METTL3 or METTL14 expression was changed. As shown in Figures 5A,B, the mRNA levels of MNK2 were not changed, while the protein levels of MNK2 were greatly upregulated when METTL3 or METTL14 was overexpressed. Accordingly, MNK2 protein levels were downregulated, while its mRNA was not affected, when METTL3 and METTL14 were knocked down (Figures 5C,D), suggesting that MNK2 protein levels were regulated by METTL3/METTL14. To assay the functional importance of MNK2, we used CRISPR inhibition to knock down MNK2 in C2C12 cells. Real-time PCR results showed that MNK2 mRNA was significantly inhibited (Figure 5E). Western blot results also confirmed that MNK2 protein expression was greatly repressed (Figure 5F). Interestingly, knocking down MNK2 decreased ERK phosphorylation levels and increased MHC protein levels (Figures 5G,H), suggesting that MNK2 could be a functional downstream target of METTL3 and METTL14 and that it inhibited C2C12 differentiation probably via ERK signaling.

METTL3 and METTL14 are known as “writers” and are critical for m^6^A deposition. Some specific RNA-binding proteins, including YTHDF1/2/3 and YTHDC1, known as “readers,” can bind m^6^A motifs to regulate corresponding RNA functions and thus induce the acquisition of specific phenotypic outcomes. To identify potential m^6^A readers for MNK2, we knocked down YTHDF1/2/3 and YTHDC1 using siRNAs and then detected the expression level of MNK2. The potential readers were successfully knocked down, as indicated by the significant decrease in their mRNA levels (Figure 5I), which did not affect MNK2 mRNA expression (Figure 5J). MNK2 protein levels were decreased only when YTHDF1 expression was knocked down, while knocking down YTHDF2/3 and YTHDC1 had no effect on MNK2 protein levels (Figure 5K), suggesting that YTHDF1 is an important reader of MNK2. The mRNA levels of MHC and MEF2C were significantly upregulated when YTHDF1 expression was knocked down (Figure 5L), indicating that YTHDF1 can repress the differentiation of C2C12 cells and is a phenotypically relevant reader for MNK2.

### METTL3/14-MNK2 Axis Is Activated During Acute Skeletal Muscle Injury

**FIGURE 6:**
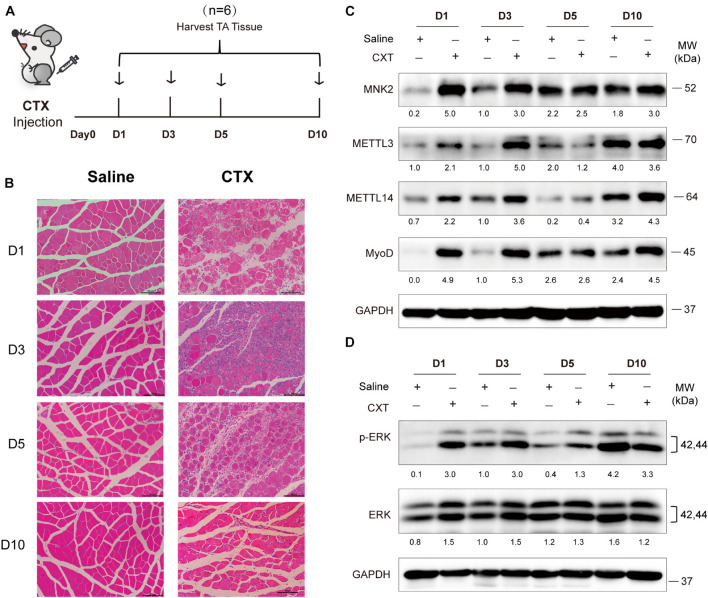
METTL3/14 are ectopically expressed during acute skeletal muscle injury, and METTL3/14-MNK2-ERK might facilitate the early stage of skeletal muscle regeneration. **(A)** Time points of mice tibialis anterior (TA) muscles injection with CTX and collected tissues on day1 (D1), day3 (D3), day5 (D5), and day10 (D10). **(B)** Hematoxylin and eosin staining of regenerated muscle on indicated recover time points. **(C)** Western blot assay of MNK2, METTL3, METTL14 and MyoD in regenerated muscle on indicated recover time points. **(D)** Western blot assay of p-ERK and ERK in regenerated muscle on indicated recover time points.

To further explore *in vivo* functions of METTL3/14, we examined the expression profiles and function of METTL3/14 in acute skeletal muscle regeneration after cardiotoxin (CTX) injection. Adult muscle regeneration is sustained by infiltrating macrophages and the consequent activation of MuSCs. During the reparative process, immune cells, especially the macrophages, infiltrate damaged skeletal muscles to release cytokines, chemokines and growth factors into the localized area that alter the micro-environment to clear cellular debris and support the regeneration through MuSCs activation (Saini et al., 2016; Shang et al., 2020). We treated mice with CTX and collected tissues at different time points (Figure 6A). As determined by hematoxylin and eosin staining of muscle, extensive necrosis post-CTX treatment was observed, and the mouse muscle was markedly regenerated 5 days after the injection, suggesting that the injury and regeneration model was successfully established (Figure 6B). At early time points after injury (day1 and day3), MyoD were upregulated, indicating that MuSCs were actively proliferating and starting differentiation. Interestingly, METTL3 and METTL4 expression, along with that of their downstream target MNK2 and p-ERK were markedly upregulated on these time points (Figures 6C,D), suggesting that METTL3/14-MNK2 may be involved in the early stage of muscle regeneration and enhance the activation and proliferation of these MuSCs.

## Discussion

**FIGURE 7:**
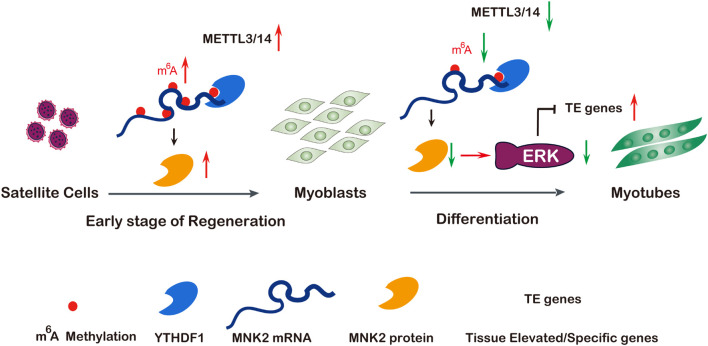
A model of METTL3/14 target MNK2 via m^6^A modification and then modulate ERK signaling pathway during skeletal muscle differentiation and regeneration. Tissue renewal and muscle regeneration largely rely on the activation and proliferation of muscular satellite cells, the expression of METTL3 and METTL14 is increased on the early stage of regeneration after injury, then promotes MNK2 expression and favors to accelerate regeneration. In differentiating myoblasts, m^6^A modification and core methyltransferases METTL3 and METTL14 are down-regulated, METTL3/14 target MNK2 directly then regulate ERK signaling to block myogenesis, so decreased expression of METTL3/14 is essential for skeletal muscle differentiation. Additionally, the m^6^A modified Mnk2 is recognized by YTHDF1.

Increasing evidence has shown that the m^6^A methylation modification may play pivotal physiological functions in various biological processes by regulating RNA metabolism (Roundtree et al., 2017; Wang et al., 2020). In this study, we showed that global m^6^A levels decreased during skeletal muscle differentiation, which was consistent with previous findings (Gheller et al., 2020). We demonstrated that METTL3 and METTL14 played critical roles in both normal myogenesis and regeneration after injury, describing a novel regulatory network of METTL3 and METTL14 modulating muscle function by positively regulating the expression of MNK2 through m^6^A-based posttranscriptional regulation, which was mediated by YTHDF1, a potential reader of m^6^A-Mnk2. Briefly, in the early stage of muscle regeneration after injury, METTL3, METTL14 and MNK2 expression were elevated, which led to enhanced self-renewal/proliferation of MuSCs for favoring regeneration. While, in normal myoblasts, METTL3 and METTL14 were highly expressed, MNK2 expression was maintained at a high level, and during skeletal muscle differentiation, METTL3 and METTL14 expression, as well as the m^6^A modification level, were decreased, thus MNK2 expression was repressed, reducing the activity of the downstream ERK/MAPK signaling pathway, and facilitating the differentiation process (Figure 7).

MNK2 has been reported to be a key phosphorylation factor in the MAPK signaling pathway (Maimon et al., 2014), and it is directly phosphorylated and activated by ERK or P38/MAPK kinase and implicated in the regulation of protein synthesis through its phosphorylation of eukaryotic translation initiation factors 4E and 4G (Eif4E, Eif4G) (Waskiewicz et al., 1997; Ueda et al., 2004). The role of MNKs in the development and progression of solid tumors and hematological malignancies has been widely discussed, particularly in the context of cap dependent translation, regulated by phosphorylation of eIF4E (Beggs et al., 2015; Wu et al., 2016). In contrast, MNK2 can also target ERK. Mnk2 binds to phosphorylated, active ERK in the C-terminus to sites within the MAPK-binding motif, and this binding apparently protects ERK against dephosphorylation and inactivation (Parra et al., 2005). Proper expression of these molecules is essential for muscle homeostasis maintenance, and a high level of MNK2 causes muscle atrophy (Hu et al., 2012). We focused our study on the role of m^6^A-modified Mnk2. Our results showed that MNK2 was positively regulated by METTL3 via m^6^A modification at the posttranslational level, leading to increased MNK2 protein expression and stimulating the ERK signaling pathway. This was somewhat surprising because most previous studies had demonstrated that MNKs were regulated by MAPKs, including P38 and ERK, which might have suggested a positive role for these kinases in translation and activity-promoting signaling.

With advances in MeRIP-seq technology, the basic features of m^6^A modification have been characterized in an increasing number of mammalian tissues and cell lines. Our m^6^A-seq data are consistent with previous findings showing that global m^6^A levels decrease during skeletal muscle differentiation (Gheller et al., 2020). Notably, the majority of m^6^A genes showed no dramatic changes at the RNA expression level in the skeletal myogenesis system in this study. For instance, Mnk2 exhibited a marked decrease in the m^6^A signal upon differentiation, and its RNA expression level was changed slightly, resulting in fewer translated proteins and facilitating muscle differentiation. This effect was reversed by overexpression of METTL3. These findings were in agreement with our recent report showing that METTL3 influenced muscle-specific miRNAs at both the transcriptional and posttranscriptional levels to block the myogenesis process (Diao et al., 2021a). In addition, we showed that YTHDF1-mediated translation was likely critical for m^6^A-modified MNK2 in muscle cells. It will be very interesting to elucidate the underlying mechanisms in the future. These results will provide clues for further functional studies of m^6^A modification.

In this study, we demonstrated a dynamic pattern of METTL3 and METTL14 expression during the shift of myoblasts to myofibers, as well as in the early stage of skeletal muscle regeneration after injury. Mettl3 and Mettl14 may have cooperative functions through their structural basis, but opposite conclusions have been reported for these two transferases in various studies (Kudou et al., 2017; Gheller et al., 2020; Zhang X. et al., 2020). To confirm our hypothesis, we overexpressed Mettl3, Mettl14 and Mettl14 in C2C12 myoblasts and MuSCs. Our results showed that these two overexpressed cell lines had a repressive effect on the differentiation process, compared to the control GFP cells. This finding was consistent with a study reporting that reducing the levels of METTL3 could force C2C12 myoblasts to prematurely differentiate, which we were studying in this project. In particular, our study provides the comprehensive description of METTL3 and METTL14 functions during myogenesis. Interestingly, METTL3/14-MNK2 appeared to promote the early stage of MuSCs proliferation, which was consistent with the recently report that METTL3 knockout in muscle stem cells significantly inhibits the proliferation of muscle stem cells and blocks the muscle regeneration after injury and konckin of METLL3 in muscle stem cells promotes the muscle stem cell proliferation and muscle regeneration *in vivo* (Liang et al., 2021). However, the underlying regulation of this mechanism remains elusive.

Skeletal muscle attenuation and defects in muscle regeneration and repair after injury are important health problems in aging societies worldwide, and there is a lack of effective prevention and treatment strategies. The regulatory mechanism of m^6^A methylation and its key methyltransferases METLL3 and METTL14 in the regeneration of skeletal muscle is still largely unknown (Zhang W. et al., 2020). In this study, we clarify the mechanism of m^6^A epigenetic regulation in differentiation and regeneration processes. Furthermore, it is worth exploring the function of the m^6^A-modified MNK2 gene in clinical muscle injury samples to provide new ideas for clinical diagnosis and treatment.

## Data Availability Statement

The original contributions presented in the study are publicly available. This data can be found here: GEO at NCBI (GSE180431). They can be accessed via https://www.ncbi.nlm.nih.gov/geo/query/acc.cgi?acc=GSE180431.

## Ethics Statement

The animal study was reviewed and approved by the Animal Ethics Committee at the Third Affiliated Hospital of Sun Yat-sen University.

## Author Contributions

S-JX, Z-DX, QZ, and Y-WP conceived and designed the experiments. S-JX, HL, and L-TD performed the experiments with the help of ST, Y-RH, Y-JS, and Y-XH. S-JX, BY, J-YL, and J-HH analyzed the data. S-JX and Z-DX wrote the manuscript. All authors contributed to the article and approved the submitted version.

## Conflict of Interest

The authors declare that the research was conducted in the absence of any commercial or financial relationships that could be construed as a potential conflict of interest.

## Publisher’s Note

All claims expressed in this article are solely those of the authors and do not necessarily represent those of their affiliated organizations, or those of the publisher, the editors and the reviewers. Any product that may be evaluated in this article, or claim that may be made by its manufacturer, is not guaranteed or endorsed by the publisher.

## Footnotes

1
https://github.com/OpenGene/fastp

2
http://daehwankimlab.github.io/hisat2/

3
https://bioconductor.org/packages/exomePeak/

4
http://www.igv.org/

5
http://homer.ucsd.edu/homer/motif/
