# Supplementary material for: Dynamic m6A mRNA Methylation Reveals the Role of METTL3/14-m6A-MNK2-ERK Signaling Axis in Skeletal Muscle Differentiation and Regeneration
Source: Front Cell Dev Biol. 2021 Oct 1;9:744171. doi: 10.3389/fcell.2021.744171 (PMC8517268; doi:10.3389/fcell.2021.744171)
Supplement: Supplementary file 1 [file Data_Sheet_1.docx]

**Supplementary Information**

Attached to Manuscript

Xie *et al*. “Dynamic m^6^A mRNA methylation reveals the role of METTL3/14-m^6^A-MNK2-ERK signaling axis in skeletal muscle differentiation and regeneration”

**Titles and Legends to Supplementary Figures**

**Figure S1.** **m^6^A methylation levels and expression levels of its key factors during C2C12 cells differentitaion.**

**(A)** Relative m^6^A levels of RNA dot blot assay of C2C12 myoblasts during differentiation. **(B)** Real-time PCR analysis of m^6^A methyltransferases METTL3, METTL14 and WTAP expression in differentiating C2C12 cells. **(C)** Western blot analysis of METTL3, METTL14 and WTAP in the primary mouse skeletal muscle cells. **(D)** Western blot analysis of FTO and ALKBH5 expression of differentiation C2C12 myoblasts. (**E**) Real-time PCR analysis of m^6^A methyltransferases METTL3, METTL14 and WTAP expression during developing mouse embryos muscle. Mouse hind limb muscles were isolated from seven time points: E12.5, E15.5, E18.5 (embryos), postnatal 1 week, postnatal 2 weeks, postnatal 4 weeks, and postnatal 8 weeks (adult). Quantitative data was represented as Mean±SD. The statistical signiﬁcance of difference between two means was calculated with the t-*test*, ***p* < 0.01, *****p*< 0.001, *****p*< 0.0001.

**Figure S2.** **Overexpression of METTL3/14 blocks differentiation of C2C12 cells.**

**(A)** Western blot analysis of MHC, GFP and METTL3 protein expression in METTL3- overexpressing C2C12 cells upon undifferentiation and differentiation for the indicated days. **(B)** Western blot analysis of MHC and METTL14 protein expression in METTL14-overexpressing C2C12 cells upon undifferentiation and differentiation for the indicated days. **(C)** Real-time PCR analysis of MHC, MEF2C and METTL3 expression in METTL3-overexpressing C2C12 cells upon differentiation for the indicated days. **(D)** Real-time PCR analysis of MHC, MEF2C and METTL14 expression in METTL14-overexpressing C2C12 cells upon differentiation for the indicated days.

**Figure S3.** **Real-time PCR results reveal overexpression of METTL3/14 blocks differentiation of MuSC cells.**

**(A)** Real-time PCR analysis of MHC, MEF2C and METTL3 expression in METTL3-overexpressing MuSCs upon differentiation for the indicated days. **(B)** Real-time PCR analysis of MHC, MEF2C and METTL14 expression in METTL14-overexpressing MuSCs upon differentiation for the indicated days.

**Figure S4. AKT signaling does not participate in METTL3/14-mediated skeletal muscle differentiation.**

**(A)** Western blot analysis of p-AKT (Thr308) and p-AKT (Ser473) protein expression of differentiation C2C12 myoblasts. **(B)** Western blot analysis of p-AKT (Thr308) and p-AKT (Ser473) protein expression in METTL3-overexpressing or knockdown C2C12 cells. **(C)** Western blot analysis of p-AKT (Thr308) and p-AKT (Ser473) protein expression in METTL14-overexpressing or knockdown C2C12 cells.

**Figure S5.** **Inhibition of ERK signaling accelerates differentiation of C2C12 cells.**

Real-time PCR analysis of MHC and MEF2C expression in C2C12 cells that were treated with different dose of PD0325901 (PD) or DMSO for 48h in growth medium (D0) or for 48h in growth medium then shifted to differentiation medium for 3 days (D3). DMSO as negative control.

**Figure S6.** **Overview of m^6^A peaks.**

**(A)** Cumulative frequency of RNA log2FC for transcripts containing m6A upon C2C12 differentiation. The pie chart shows m^6^A peaks distribution ration at 5’UTR, CDS, 3’UTR regions and ncRNA of undifferentiated myoblasts (GM) **(B)** or myoblasts differentiated for 4 days (D4) **(C)**.

**Titles to Supplementary Tables**

**Suppl. Table S1.** **The sequences of primers used in this study.**

**Suppl. Table S2.** **The sequences of siRNAs used in this study.**

**Suppl. Table S3. Overview of** **RNA-seq reads in this study.**

**Table S4.** **Differentially expressed genes in METTL3-overexpressing cells compared to the control GFP-overexpressing cells.**

The differentially expressed genes were identified using DESeq2 package, genes with FDR(false discovery rate)≤ 0.05 [were](file:///G:\107.研究课题\107-1.%20m6A\107-1-20.%20NAR\NAR-0514\were) considered statistically significant.

**Suppl. Table S5.** **Differentially expressed genes in METTL14-overexpressing cells compared to the control GFP-overexpressing cells.**

The differentially expressed genes were identified using DESeq2 package, genes with FDR(false discovery rate)≤ 0.05 [were](file:///G:\107.研究课题\107-1.%20m6A\107-1-20.%20NAR\NAR-0514\were) considered statistically significant.

**Suppl. Table S6.** **KEGG pathway analysis of the differentially expressed genes in METTL3-overexpressing cells compared to the control GFP-overexpressing cells.**

**Suppl. Table S7.** **KEGG pathway analysis of the differentially expressed genes in METTL14-overexpressing cells compared to the control GFP-overexpressing cells.**

**Suppl. Table S8. Overview of MeRIP-seq reads in this study.**

**Suppl. Table S9.** **m^6^A unique peaks from MeRIP-seq sequencing library of GM.**

C2C12 cells that were cultured in growth medium.

**Suppl. Table S10.** **m^6^A unique peaks from MeRIP-seq sequencing library of D4.**

C2C12 cells that were cultured in growth medium then shifted into differentiation medium for 4 days.

**Suppl. Table S11.** **Differentially m^6^A unique peaks in D4 cells compared to the GM cells.**

The differentially expressed genes were identified using DESeq2 package, genes with FDR(false discovery rate)≤ 0.05 [were](file:///G:\107.研究课题\107-1.%20m6A\107-1-20.%20NAR\NAR-0514\were) considered statistically significant.

**Suppl. Table S12.** **KEGG pathway analysis of the differentially expressed m^6^A genes in D4 cells compared to the GM cells.**
