# Supplementary figures and images for: Dynamic m6A mRNA Methylation Reveals the Role of METTL3/14-m6A-MNK2-ERK Signaling Axis in Skeletal Muscle Differentiation and Regeneration
Source: Front Cell Dev Biol. 2021 Oct 1;9:744171. doi: 10.3389/fcell.2021.744171 (PMC8517268; doi:10.3389/fcell.2021.744171)

Figure S1

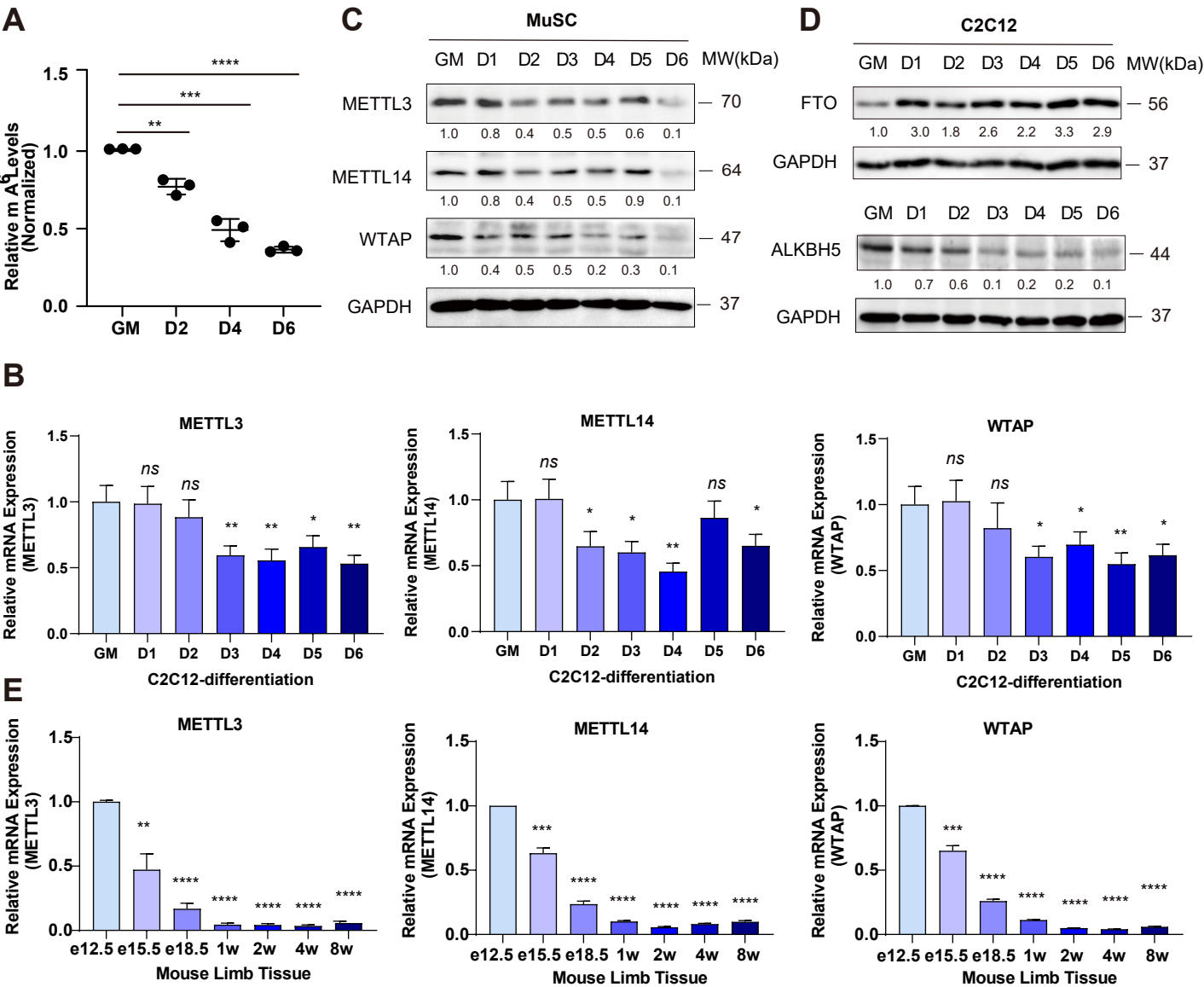

Figure S2

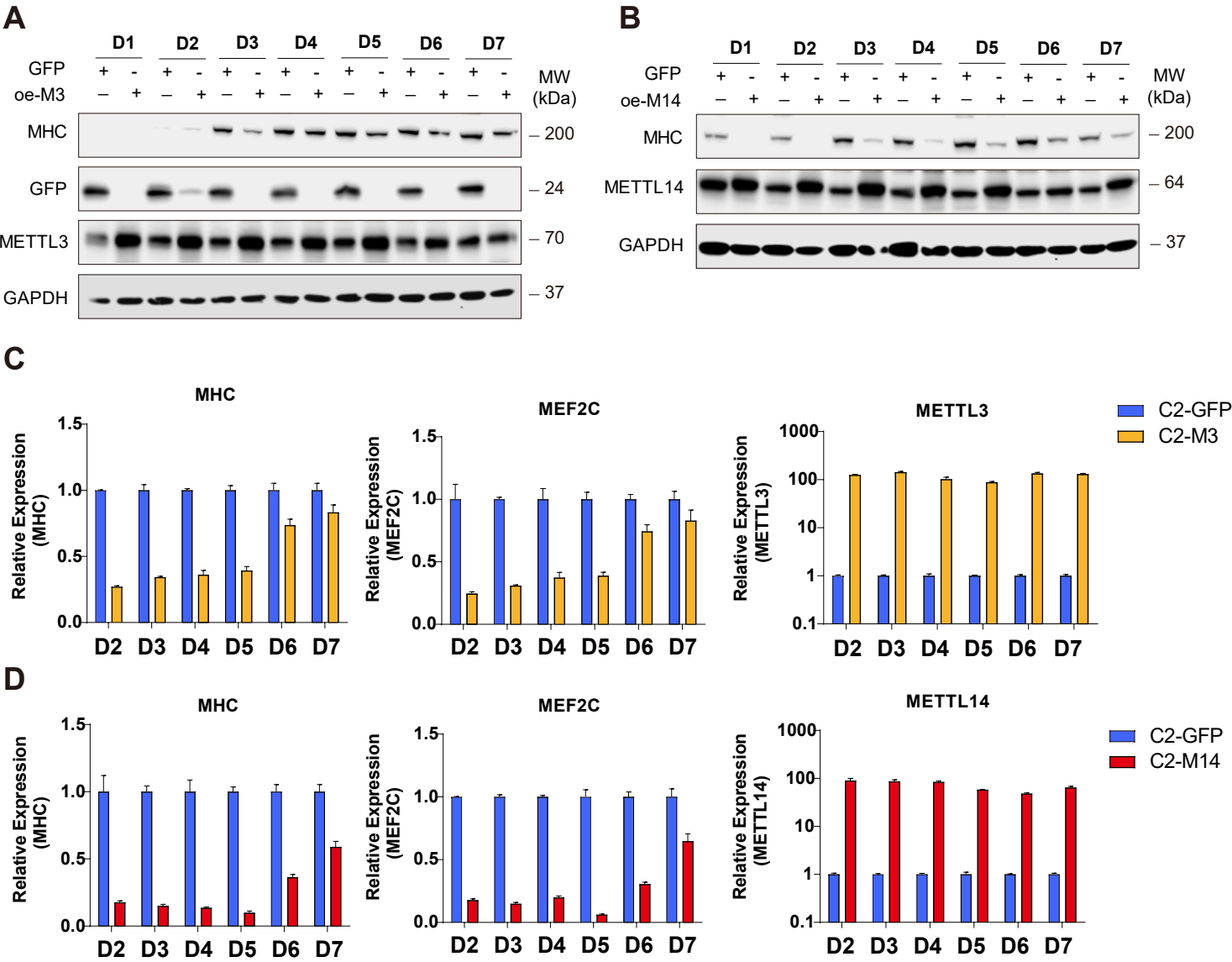

Figure S3

A

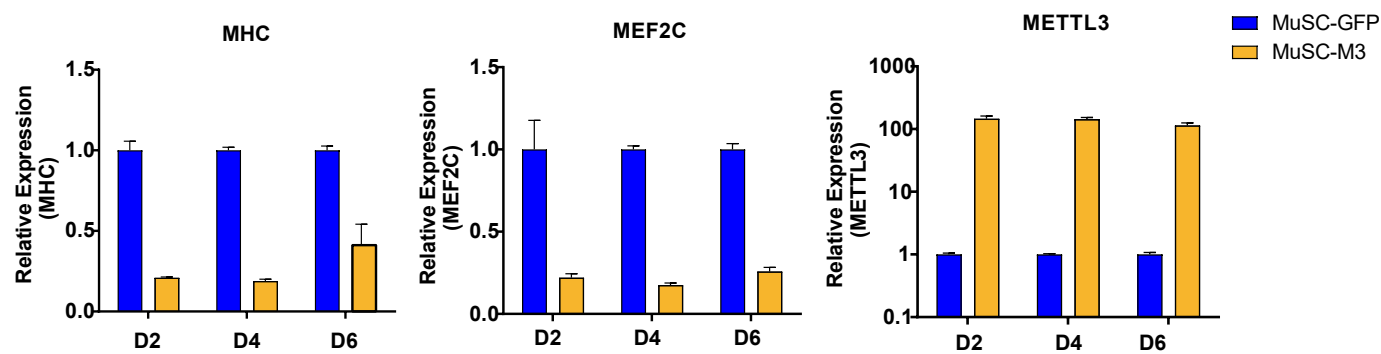

B

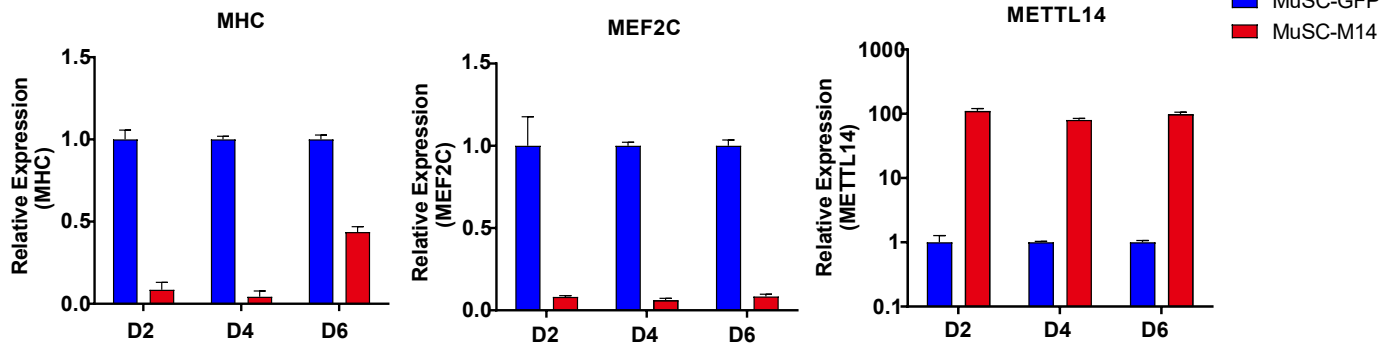

**Figure S4**

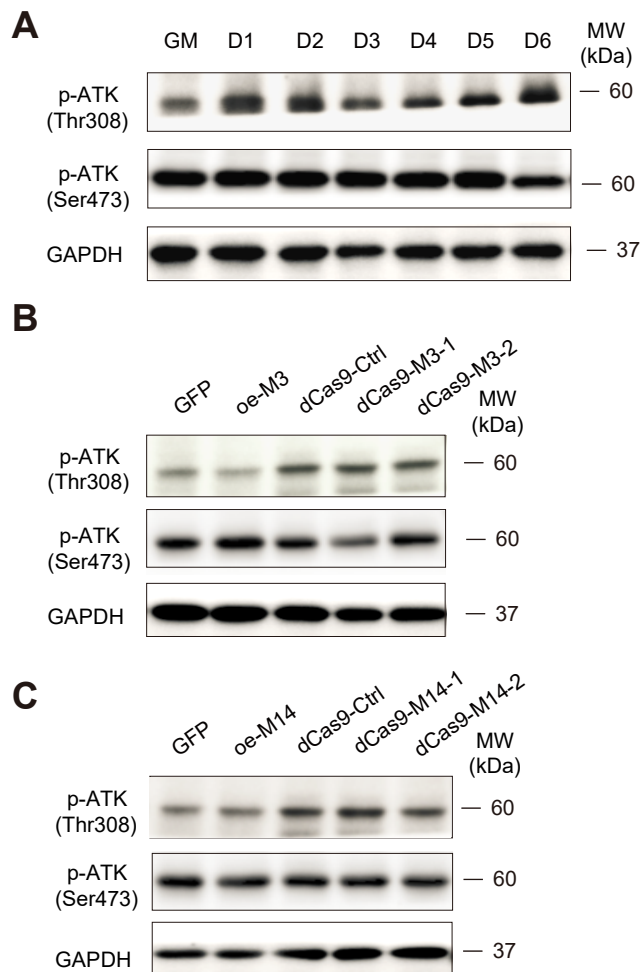

Figure S5

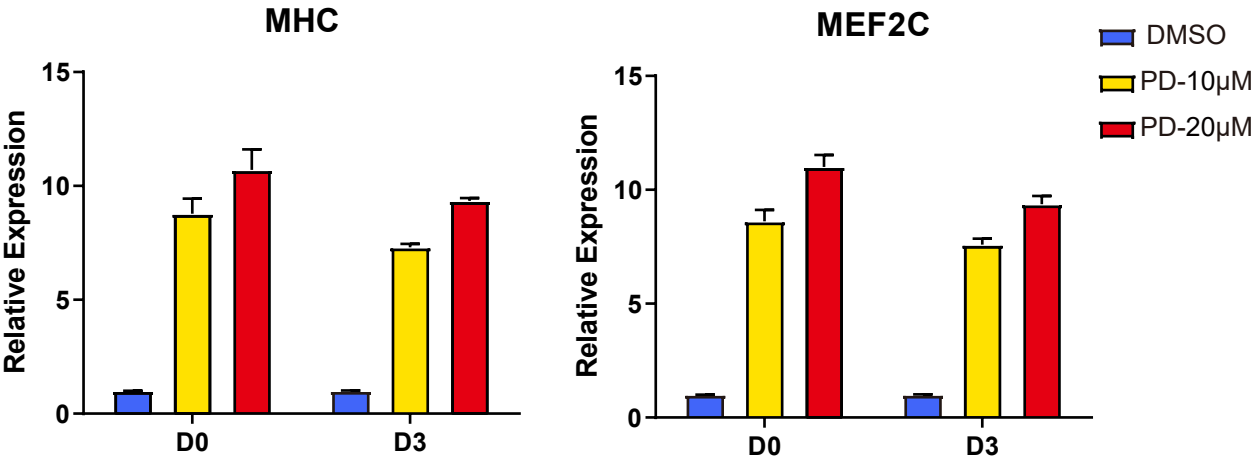

Figure S6

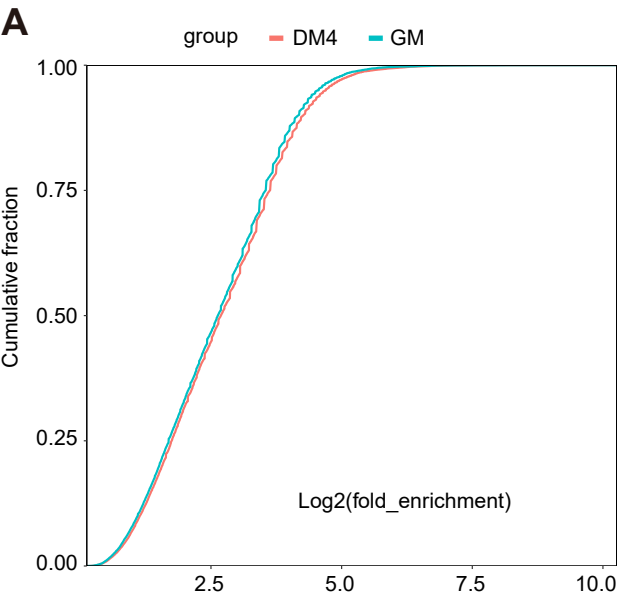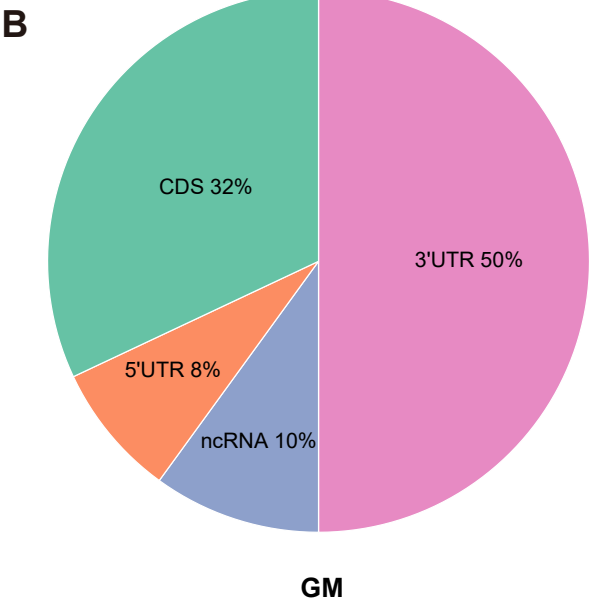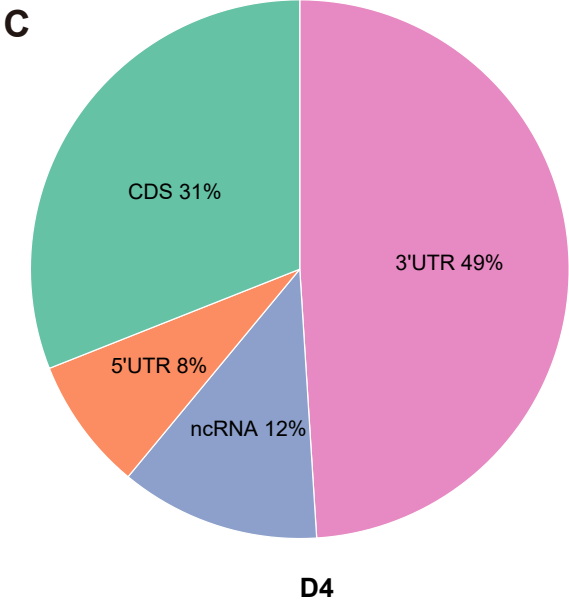

Supplement: Supplementary file 2 [file Presentation_1.pdf]
